# Enhanced neural synchrony between left auditory and premotor cortex is associated with successful phonetic categorization

**DOI:** 10.3389/fpsyg.2014.00394

**Published:** 2014-05-06

**Authors:** Jussi Alho, Fa-Hsuan Lin, Marc Sato, Hannu Tiitinen, Mikko Sams, Iiro P. Jääskeläinen

**Affiliations:** ^1^Brain and Mind Laboratory, Department of Biomedical Engineering and Computational Science (BECS), School of Science, Aalto UniversityEspoo, Finland; ^2^Institute of Biomedical Engineering, National Taiwan UniversityTaipei, Taiwan; ^3^Gipsa-Lab, Department of Speech and Cognition, French National Center for Scientific Research and Grenoble UniversityGrenoble, France; ^4^MEG Core, Aalto NeuroImaging, School of Science, Aalto UniversityEspoo, Finland; ^5^AMI Centre, Aalto NeuroImaging, School of Science, Aalto UniversityEspoo, Finland

**Keywords:** magnetoencephalography, MEG, speech perception, dorsal stream, sensorimotor integration, premotor cortex

## Abstract

The cortical dorsal auditory stream has been proposed to mediate mapping between auditory and articulatory-motor representations in speech processing. Whether this sensorimotor integration contributes to speech perception remains an open question. Here, magnetoencephalography was used to examine connectivity between auditory and motor areas while subjects were performing a sensorimotor task involving speech sound identification and overt repetition. Functional connectivity was estimated with inter-areal phase synchrony of electromagnetic oscillations. Structural equation modeling was applied to determine the direction of information flow. Compared to passive listening, engagement in the sensorimotor task enhanced connectivity within 200 ms after sound onset bilaterally between the temporoparietal junction (TPJ) and ventral premotor cortex (vPMC), with the left-hemisphere connection showing directionality from vPMC to TPJ. Passive listening to noisy speech elicited stronger connectivity than clear speech between left auditory cortex (AC) and vPMC at ~100 ms, and between left TPJ and dorsal premotor cortex (dPMC) at ~200 ms. Information flow was estimated from AC to vPMC and from dPMC to TPJ. Connectivity strength among the left AC, vPMC, and TPJ correlated positively with the identification of speech sounds within 150 ms after sound onset, with information flowing from AC to TPJ, from AC to vPMC, and from vPMC to TPJ. Taken together, these findings suggest that sensorimotor integration mediates the categorization of incoming speech sounds through reciprocal auditory-to-motor and motor-to-auditory projections.

## INTRODUCTION

Current theories propose that speech is cortically processed by the ventral and dorsal auditory streams (Hickok and Poeppel, 2007; Rauschecker and Scott, 2009). While the ventral stream processes acoustic-phonetic features of speech, the dorsal stream has been suggested to mediate mapping between auditory and articulatory-motor representations (Hickok et al., 2011; Rauschecker, 2011). Whether this sensorimotor integration contributes to the perception of others’ speech remains debated (Cappa and Pulvermuller, 2012; Hickok, 2012; Schwartz et al., 2012).

As the speech signal has high variability and complex composition of acoustic features, it has been suggested that the listener’s internal articulatory knowledge might be important in the categorization of incoming speech sounds (Liberman et al., 1967; Liberman and Mattingly, 1985; Davis and Johnsrude, 2007; Schwartz et al., 2012). Experimental support for such motor contribution is provided by findings showing that disturbing the left premotor cortex (PMC) or lip/tongue areas in the primary motor cortex (MC) with transcranial magnetic stimulation (TMS) results in impaired speech sound identification and discrimination (Meister et al., 2007; Möttönen andWatkins, 2009; Sato et al., 2009; D’Ausilio et al., 2011; Grabski et al., 2013). Möttönen et al. (2013) further demonstrated that the TMS-induced disruption of articulatory-motor cortex impairs also automatic speech sound discrimination (i.e., in the absence of behavioral tasks and without explicit attention directed to the speech sounds). In a related study, Chevillet et al. (2013) observed, using a functional magnetic resonance imaging (fMRI) adaptation paradigm, automatic phoneme category selectivity in the left PMC that correlated positively with behavioral categorization performance.

Further supporting the sensorimotor nature of speech perception, a study applying concurrent magnetoencephalography (MEG) and electroencephalography (EEG) with Granger causation analyzes found that activation in the posterior superior temporal gyrus (pSTG) was influenced by activation in dorsal PMC (dPMC) during perception of coarticulated speech, thus suggesting that articulatory processes directly mediate speech perception (Gow and Segawa, 2009). An fMRI study demonstrated that speech motor areas, in particular the ventral PMC (vPMC), were more strongly activated by non-native compared to native phonemes, which can be interpreted as being caused by the motor system repeatedly iterating in order to find the best match for the unfamiliar acoustic input among candidate phonemic categorizations (Wilson and Iacoboni, 2006). A similar process can be expected in case of degraded native speech, as it has been shown that degraded compared to clear speech elicits enhanced responses in motor areas, including the inferior frontal gyrus (IFG) and PMC (e.g., Davis and Johnsrude, 2003). Relatedly, simultaneous MEG and EEG recordings demonstrated that perceptual clarity of degraded speech was enhanced by prior knowledge of speech content and associated with activity in the IFG that preceded activity changes in the STG, therefore suggesting that prior knowledge is integrated with speech inputs through top-down predictions from the speech motor areas to lower-level sensory cortex (Sohoglu et al., 2012).

Compatible with these studies, our recent MEG study with minimum-norm estimate (MNE) -based source modeling showed that activity in the left PMC was amplified at ~200 ms after sound onset when subjects were to identify and repeat the presented speech sound compared to passive listening, with the effect being stronger when the sounds were masked by acoustic noise compared to clear speech (Alho et al., 2012). Also, the left PMC activity at ~100 ms after sound onset correlated positively with speech sound identification accuracy. However, these findings alone do not answer the question whether performance in such sensorimotor task involves reciprocal auditory-to-motor and motor-to-auditory projections, which have been hypothesized to be crucial in constraining the interpretation of incoming acoustic speech information with complementary articulatory information (Schwartz et al., 2012). According to a recent dual-pathway model of auditory cortical processing, speech sounds are processed hierarchically in the ventral stream from the auditory cortex (AC) to the category-invariant inferior frontal cortex (IFC), transformed into articulatory representations in the vPMC, and finally transmitted to the temporoparietal junction (TPJ) as an efference copy (Rauschecker and Scott, 2009; Rauschecker, 2011). In this model, processing in the dorsal stream proceeds from the AC to the TPJ, where a quick sketch of sensory event information is compared with the efference copy of the activated articulatory-motor plans. Tentatively, such sensorimotor integration could be enabled by oscillatory synchrony, i.e., rhythmic millisecond-range temporal correlations of neuronal activity (Womelsdorf et al., 2007; Singer, 2009). Previous MEG and EEG studies have revealed that the level of inter-areal phase synchrony within the alpha (8–14 Hz), beta (14–30 Hz) and gamma (30–80 Hz) frequency bands correlates with various perceptual, attention, and working memory task performances (Kujala et al., 2007; Palva et al., 2010; Hipp et al., 2011; Kveraga et al., 2011; Huang et al., 2014), therefore supporting the hypothesis that coordinated operation between task-relevant brain regions is reflected as strengthened oscillatory synchrony (for a review, see Palva and Palva, 2012).

Here, we analyzed our previously published MEG dataset (Alho et al., 2012) to estimate functional connectivity among speech-relevant brain areas while subjects were performing a sensorimotor integration task involving speech sound identification and overt repetition. We utilized the increased spatiotemporal accuracy provided by MRI-based MNEs (Lin et al., 2006) to estimate inter-areal neural synchrony. Continuous wavelet transform of single-trial data was applied to reveal the phase dynamics of ongoing neural activity as a function of time and frequency. The level of phase synchrony was quantified with weighted phase lag index (WPLI; Vinck et al., 2011). In addition, directionality of information flow was estimated with structural equation modeling (SEM; Penny et al., 2004). We hypothesized that the neural synchrony between auditory and motor areas within 200 ms after sound onset is (1) enhanced when one is engaged in the sensorimotor task compared to passive listening; (2) enhanced when the sounds are masked by acoustic noise compared to clear speech; and (3) positively correlated with the speech sound identification accuracy.

## MATERIALS AND METHODS

### SUBJECTS

Twenty-two healthy individuals with self-reported normal hearing participated in the study. Two subjects were excluded from the analyses due to low signal-to-noise ratio (SNR), resulting in a final sample size of 20 subjects (18 right-handed, age range 21–58 years, mean ± SD age: 27.4 ± 8.0 years). All except one (Italian) were native speakers of Finnish. Informed consent was obtained from all subjects. The experiment was approved by the Coordinating Ethics Committee of the Hospital District of Helsinki and Uusimaa.

### STIMULI AND TASK

The stimuli were /pa/ and /ta/ syllable sounds articulated by a male native Finnish speaker and presented either as intact or embedded in noise. Five individual clearly articulated /pa/ and /ta/ tokens were selected, scaled to 68 dB, and cut at 100 ms preceding and following the detected consonantal burst. Thus, the duration of the spoken syllable was 100 ms. Noisy speech stimuli were created by masking the syllables with Gaussian pink noise. The masks had a 5-ms rise-decay envelope, were de-emphasized to better match the frequency spectrum of /pa/ and /ta/ syllables (at -6 dB/oct), and were simultaneously presented from the beginning to the end of the syllable with SNR of + 5 dB. A forced-choice identification test with a subset of six subjects was conducted to ensure appropriate syllable identification accuracy at this SNR level (i.e., 77% correct responses).

The stimuli were presented in four different conditions: passive perception; perception followed by overt repetition; perception followed by covert repetition; and perception followed by overt imitation. In the active conditions, the subjects’ task was to identify the syllable as either /pa/ or /ta/, wait for a visual cue, and reproduce it accordingly. The overt imitation task differed from the overt repetition in that the reproduction of the target syllable was to be done by imitating the pitch of the stimulus sound. The covert repetition was to take place covertly without any articulatory movements or sound production.

**FIGURE 1:**
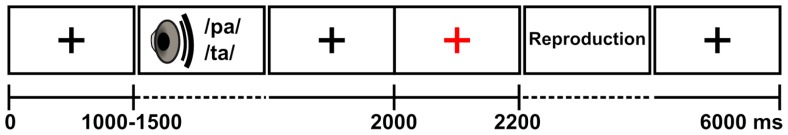
**Experimental procedure**. Adapted from Alho et al. (2012).

Each condition comprised 300 trials (75 intact /pa/ + 75 intact /ta/ + 75 noisy /pa/ + 75 noisy /ta/) presented with (1) a randomly varying 1–1.5 s prestimulus baseline for perception, (2) randomized auditory stimulus presentation (/pa/ or /ta/), (3) a baseline for repetition of the syllable (300–800 ms after stimulus offset), and (4) a visual cue to repeat (black fixation cross turning briefly to red; 2–2.2 s). Thus, the total duration of the trial was 6 s, with interstimulus interval (ISI) varying between 5.5 and 6.5 s, and the interval between the onset of the auditory stimulus and the subsequent visual cue to repeat varying between 0.5 and 1 s (**Figure 1**). The measurement time per condition totaled to ~30 min, which was divided into two ~15 min blocks to prevent fatigue. The measurements were divided on 2 days, with the passive listening and overt repetition conditions on the first day, and covert repetition and imitation conditions on the second day. The order of the conditions was kept fixed to reduce the possibility of the performance in the less demanding tasks being affected by the experience from the more demanding tasks (e.g., to reduce the subjects’ disposition to covertly rehearse the presented stimuli in the passive listening condition or to imitate when natural repetition was required). The covert repetition and imitation conditions were not included in the analyses of the present study. The auditory stimuli were presented via a panel loudspeaker with an approximate 65-dB sound level. All stimuli were delivered with Presentation software (v10.1, Neurobehavioral systems).

### DATA RECORDING

The MEG data were acquired with a whole-head 306-channel neuromagnetometer (VectorView, Elekta-Neuromag, Finland) of the MEG Core of Aalto NeuroImaging infrastructure at Aalto University. The device was situated in a magnetically shielded room, with a three-layer μ-metal and aluminum cover to attenuate effects of outside magnetic fields, and an additional active noise-cancelation system.

Before each MEG recording session, locations of four head position indicator (HPI) coils attached to the scalp were recorded with respect to three anatomical landmark points (nasion and two preauricular points) using a 3-D digitizer (Isotrak, Polhemus, Colchester, VT, USA). Additional scalp surface points (≈30) were digitized to facilitate coregistration with anatomical magnetic resonance (MR) images. To detect eye blinks and movements, an electro-oculogram (EOG) channel was recorded with electrodes placed below and on the outer canthus of the left eye. The MEG signals were band-pass filtered at 0.03–200 Hz and digitized at a sampling frequency of 2000 Hz. The individual MR images were acquired with a 3T GE Signa scanner (GE Healthcare Ltd., Chalfont St Giles, UK) of the AMI Center of Aalto NeuroImaging infrastructure at Aalto University.

For subsequent identification of the subjects’ repetitions, microphone recordings with 22.05 kHz sampling rate together with electromyographic (EMG) channels with electrodes placed on three specific articulators (sternohyoid, orbicularis oris superior, and masseter) were recorded. The EMG responses were used also to control for the presence of any covert articulations that might have occurred after the perception of the syllables (i.e., before the onset of the cued reproduction task).

### MEG SOURCE ESTIMATION

The MEG data were processed and analyzed with the MNE software package (Gramfort et al., 2014). The data were first downsampled to 1000 Hz and screened for artifacts. Epochs from 200 ms preceding and 500 ms following the stimulus onset were processed separately for the stimulus types. Non-functioning (i.e., flat) channels and trials with the epochs exceeding 3000 fT/cm amplitude (measured with respect to a 200-ms prestimulus baseline) in the MEG channels or 150 μV in the EOG channel were rejected from further analyses, resulting in an average of ~120 trials/condition/stimulus type.

Source modeling was performed by computing MNEs (Hämäläinen and Ilmoniemi, 1994) from MRI-constrained MEG data. For this purpose, a single-compartment boundary element model (BEM; Hämäläinen and Sarvas, 1989) was constructed from the structural MRI and used as a forward model to constrain MEG source locations to the cortex. The source current strengths at each source location for each time point were estimated with the anatomically constrained linear estimation approach (Dale et al., 2000). To this end, an inverse operator was calculated with the help of a noise covariance matrix estimated from the filtered single-trial 200-ms prestimulus baselines. For visualizing the mean evoked activity on the cortical surface, dynamic statistical parametric map (dSPM) estimates were generated (Dale et al., 2000). As a measure of signal-to-noise (derived through normalizing the MNE by the noise sensitivity at each cortical location), dSPM indicates the locations with MNE amplitudes above the noise level. Since individual MRI-images were not available for six subjects, a FreeSurfer average brain was applied as a surrogate in these subjects (by aligning the individual fiducial points to the fiducial points of the average head).

### REGIONS-OF-INTEREST (ROIs)

**FIGURE 2:**
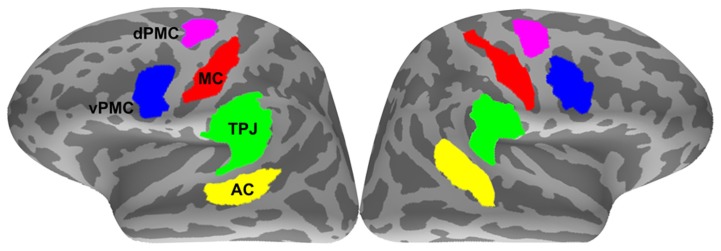
**Regions-of-interest (ROIs)**. AC, auditory cortex; TPJ, temporoparietal junction; MC, motor cortex; vPMC, ventral premotor cortex; dPMC, dorsal premotor cortex.

The inter-areal phase synchrony of the source data was investigated between ROIs. Considering that the MNE source estimation provides an underdetermined solution to the inverse problem (i.e., 306 measurement sensors to ~7000 unknown source dipoles), five large anatomical regions per hemisphere were first selected on the basis of our a priori hypothesis by merging the labels of relevant gyri and sulci that resulted from the automatic anatomical parcellation (Destrieux et al., 2010): AC (comprising the superior temporal gyrus and sulcus), TPJ (comprising supramarginal gyrus, angular gyrus, and planum temporale), pIFG/vPMC (comprising the pars opercularis of the IFG and the inferior part of the precentral sulcus), dPMC (comprising the superior part of the precentral sulcus), and MC (comprising the central sulcus). Functional constraints were then applied to these anatomical regions by selecting only the subregions where the group-average dSPM activations exceeded a threshold value of 4 (*F*-statistic) at any time between 50 and 200 ms (see Statistical analysis for the selection criteria of the analysis time window). For minimizing bias (Kriegeskorte et al., 2009), the stimulus types and conditions used for the functional constraints between different analyses were as follows: noisy stimuli in the passive listening condition for the correlation tests between neural synchrony and syllable identification accuracy; combined noisy and intact stimuli in the passive listening condition for analyzing changes in neural synchrony between noisy and clear speech; and combined noisy and intact stimuli in combined passive and active listening (i.e., overt repetition) conditions for analyzing changes in neural synchrony between passive and active listening. The ROIs were defined on the FreeSurfer average brain (**Figure 2**) and morphed onto the individual surfaces with an automatic spherical morphing procedure (Fischl et al., 1999).

### PHASE SYNCHRONY ESTIMATION

Single-trial raw (0.03–200 Hz) MNE currents from -200 to +500 ms were baseline corrected (with respect to the 200 ms prestimulus period), averaged over the source locations to obtain a time course for each ROI (by only keeping the radial components and applying sign-flips to reduce signal cancellations), and submitted to the phase synchrony analysis. Trials counts between conditions were equalized for reducing bias.

Phase synchrony between ROIs was estimated by computing a WPLI (Vinck et al., 2011) across trials for every time and frequency point. WPLI was chosen as a measure for its low sensitivity to the volume conductor effect (i.e., artificial synchrony caused by mixing of neuronal signals). This attribute is based on the idea that non-zero phase lag between two time courses is not caused by volume conduction from a common source, but rather by actual communication between brain structures through a physical medium, which is bound to have a delay (or a non-zero phase lag). The WPLIs were obtained by first filtering the ROI time courses with a continuous Morlet wavelet transform into 25 center frequencies from 8–80 Hz with 3 Hz steps (wavelet width varying from 1.1 at lowest frequency to 11.4 cycles at highest frequency). The non-zero phase lag interdependencies were then estimated, for a particular frequency, by weighting the contribution of observed phase leads and lags by the magnitude of the imaginary component of the cross-spectrum between each pair of ROIs (Vinck et al., 2011). WPLI-values range from 0 to 1, with 0 indicating random distribution of phase and 1 indicating constant (non-zero lag) phase difference across trials.

#### Statistical analysis

Spearman rank correlation test was applied to examine correlations between neural synchrony and syllable identification accuracy. For assessing changes in neural synchrony between active and passive listening, and their interaction with noisy vs. clear speech, a two-way repeated measures analysis of variance (ANOVA) was conducted. Changes in neural synchrony between noisy and clear speech was analyzed with one-way ANOVA in the passive condition to avoid the possible confounding effect caused by subjects covertly rehearsing the presented syllable while waiting for the visual cue in the active listening condition. As it has been shown that acoustic-phonetic features of speech modulate auditory cortical activity from 50 ms onwards and that the access to phonological categories occurs at ~150 ms after stimulus onset (for a review, see Salmelin, 2007), a time range of 50–200 ms was selected for the analyses. Restricting the analysis to early latencies also decreases the likelihood that the phase synchrony effects might be due to speech preparation after subjects have identified the auditory target. Within the analysis range, the WPLIs were averaged into 10-ms time windows. The *p*-values were FDR-corrected for multiple ROI connection × time × frequency point comparisons (Benjamini et al., 2001).

To control for the possibility that the phase synchrony effects could be explained by the regions independently synchronizing to the stimulus onset (i.e., phase resetting by stimulus-evoked responses) a surrogate data was created by adopting a trial shuffle approach (Lachaux et al., 1999). One thousand artificial trial orders were generated by randomly shuffling the trials in each ROI independently. For each randomization, WPLIs were calculated as described in Section “Phase Synchrony Estimation”. A *p*-value was acquired by determining the percentage of the surrogate values exceeding the original WPLI (or correlation coefficient in the correlation tests). The null hypothesis (i.e., phase synchrony results are explained by the regions independently synchronizing to the stimulus onset) was rejected at *p* < 0.05.

For estimating directionality of information flow for the significant functional connections, a *post hoc* SEM analysis was conducted (Penny et al., 2004). The SEM was performed in the same time and frequency range as the given phase synchrony effect. Continuous wavelet transform was applied to decompose the ROI time courses into time-frequency representations, similarly to the phase synchrony calculations. As samples in MEG time series are not independent, which can lead to inflated correlation between ROIs and thus bias the estimated path coefficients, the significance of the estimated paths was quantified with a bootstrap approach allowing the statistical inferences on the estimated paths to be based on empirical, rather than theoretical, estimates of the null distribution of path coefficients.

Pairwise path coefficients were tested for models with reciprocal connections between ROIs (i.e., ROI1→ROI2→ROI1). Statistical significance was tested across subjects with a paired-samples permutation *t*-test on the path coefficients (β) of the directed connections (i.e., β_A__→__B_ vs. β_B__→__A_). The goodness-of-fit between the model and data was tested with the root mean square error of approximation (RMSEA; Steiger, 1990), based on the chi-square test statistic (Pearson, 1900). A RMSEA value less than 0.07 is considered a good fit (Steiger, 2007).

All analyses and statistical tests on phase synchrony were implemented in Python, with the help of MNE-Python (Gramfort et al., 2014) and SciPy toolkit (http://www.scipy.org/). Analyses and statistical tests on SEM were implemented in MATLAB (Mathworks, Natick, MA, USA) using custom scripts and computer resources within the Aalto Science-IT project.

## RESULTS

### BEHAVIORAL RESULTS

Phonetic categorization performance was quantified as the ratio of correctly vs. incorrectly identified noisy syllables in the active listening condition involving overt repetition (/pa/ vs. /ta/; mean d-prime = 1.29, SD = 0.95; mean percent correct = 70.4%, for /pa/ 62.4%, for /ta/ 78.0%, SD = 13.6%).

### INTER-AREAL NEURAL SYNCHRONY

#### Effect of stimulus type and condition

**FIGURE 3:**
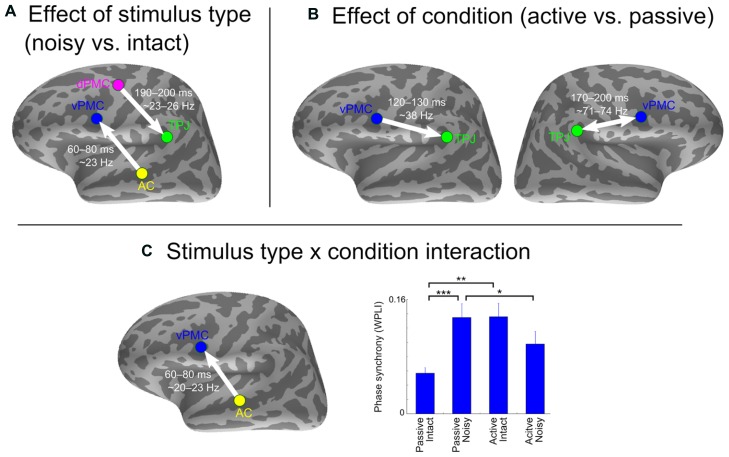
**Effect of stimulus type and condition on inter-areal phase synchrony. (A)** Stronger synchrony in response to noisy compared to intact stimulus type. **(B)** Stronger synchrony in active compared to passive listening condition. **(C)** Stimulus type x condition interaction and results from a *post hoc t*-test showing differences between conditions and stimulus types at the time-frequency point of strongest interaction. The arrows indicate SEM-derived directionality effects based on the pairwise path coefficients. The double arrow denotes undirected interaction. Asterisks indicate significant differences (*p < 0.05, **p < 0.01, ***p < 0.001, uncorrected). Error bars indicate SE.

**Figure 3** shows the effects of intelligibility (noisy vs. clear stimuli) and task (active vs. passive listening) as well as their interaction on inter-areal neural synchrony. Only the significant time-frequency points that coincided with significant values as compared to the trial-shuffled null distribution are reported.

Stronger neural synchrony was observed in response to noisy compared to intact syllables between two pairs of left-hemisphere ROIs: (1) AC and vPMC from 60–80 ms ~23 Hz [*F*(1,19) = 36.5, pFDR = 0.008]; and (2) dPMC and TPJ from 190–200 ms at ~23–26 Hz [*F*(1,19) = 34.9, pFDR = 0.02; **Figure 3A**]. The intact stimuli did not elicit stronger neural synchrony than the noisy stimuli between any pairs of ROIs.

Stronger neural synchrony was found in active compared to passive listening condition for (1) left TPJ and vPMC from 120–130 ms at ~38 Hz [*F*(1,19) = 27.1, pFDR = 0.04]; and (2) right TPJ and vPMC from 170–200 ms at ~71–74 Hz [*F*(1,19) = 43.3, pFDR = 0.001; **Figure 3B**]. None of the ROI pairs showed stronger synchrony in passive compared to active listening condition.

Significant condition x stimulus type interaction was observed between left AC and vPMC from 60–80 ms ~20–23 Hz [*F*(1,19) = 44.6, pFDR = 0.0008]. *Post hoc t*-test revealed that this was caused by stronger synchrony in response to noisy speech only in the passive listening condition (**Figure 3C**). All *F*- and *p*-values are from the time-frequency point of strongest effect.

Direction of information flow between the ROI pairs that showed significant synchrony effects was assessed using the pairwise path coefficients obtained with SEM (depicted with arrows in **Figure 3**). Directed interactions were found from left AC to vPMC [*t*(19) = 8.14, *p* < 0.001], from left dPMC to TPJ [*t*(19) = 2.78, *p* = 0.02], and from left vPMC to TPJ [*t*(19) = 3.02, *p* = 0.01]. No significant directionality was found between the right vPMC and TPJ [*t*(19) = 0.93, *p* = 0.36].

#### Correlation with speech sound identification accuracy

**FIGURE 4:**
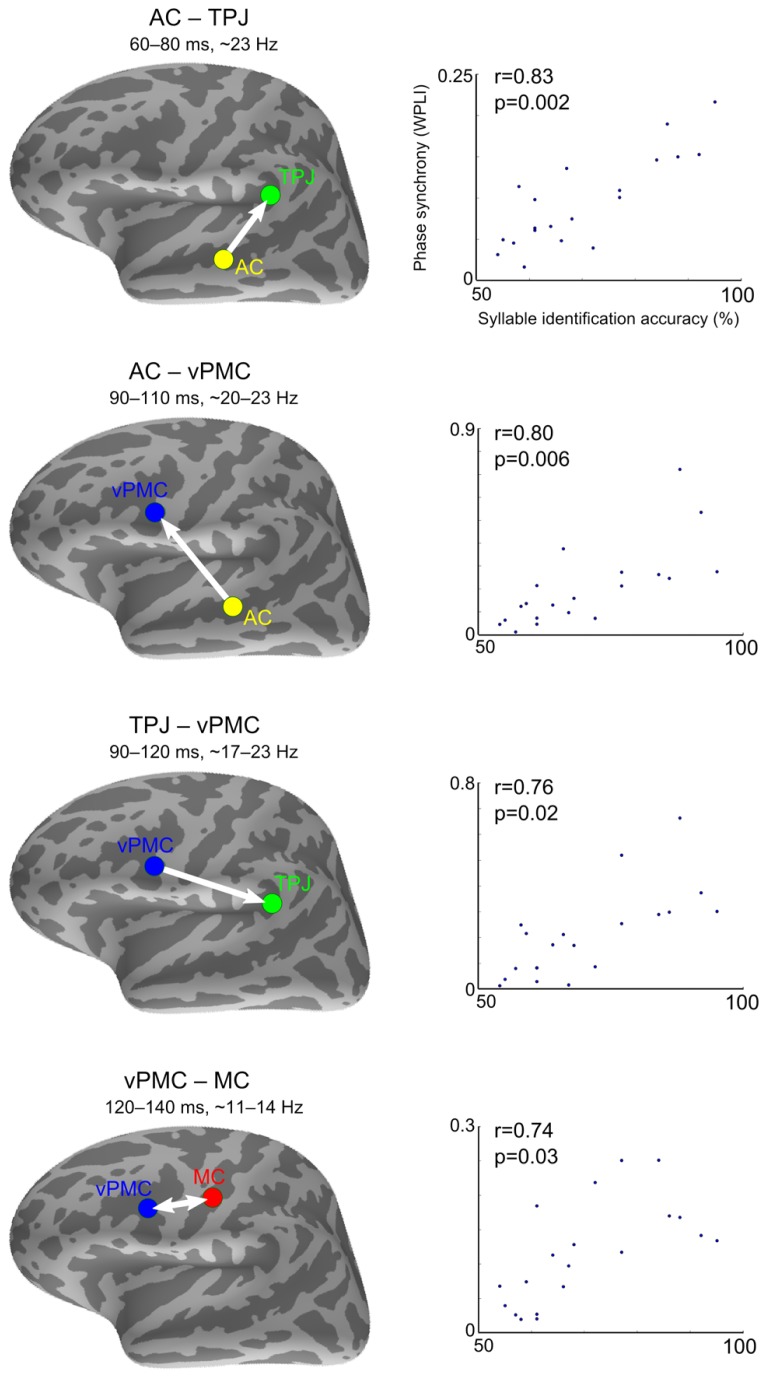
**Correlations between inter-areal phase synchrony and syllable identification accuracy**. Syllable identification scores plotted against phase synchrony strength (WPLI) at the time-frequency point of strongest correlation. The spearman rank correlation coefficients (*r*) and corresponding *p*-values are denoted in each plot. The arrows indicate SEM-derived directionality effects based on the pairwise path coefficients. The double arrow denotes undirected interaction. AC, auditory cortex; TPJ, temporoparietal junction; vPMC, ventral premotor cortex; MC, motor cortex.

As shown in **Figure 4**, speech sound identification accuracy correlated positively with four left-hemisphere connections: (1) between AC and TPJ from 60–80 ms after stimulus onset at ~23 Hz (spearman *r* = 0.83, pFDR = 0.002); (2) between AC and vPMC from 90–110 ms at ~20–23 Hz (spearman *r* = 0.80, pFDR = 0.006); (3) between TPJ and vPMC from 90–120 ms at ~17–23 Hz (spearman *r* = 0.76, pFDR = 0.02), and (4) between vPMC and MC from 120–140 ms at ~11–14 Hz (spearman *r* = 0.74, pFDR = 0.03). The correlation coefficients and *p*-values are from the time-frequency point of strongest correlation. Correlation between phase synchrony and syllable identification accuracy was not found with respect to the left dPMC or between any right-hemispheric ROIs.

The trial-shuffling analysis showed that all the phase synchrony effects remained significant after controlling for the possibility that the ROIs were independently synchronizing to the stimulus onset. The *p*-values (averaged across the significant time-frequency points) for the significance of the residual induced phase synchrony were as follows: AC–TPJ (*p* = 0.001), AC–vPMC (*p* = 0.007), TPJ–vPMC (*p* = 0.007), and vPMC–MC (*p* = 0.003). The speech sound identification performance showed no statistical outliers or correlation with subjects’ age (spearman *r* = -0.09, *p* = 0.69; age range 21–58 years, with one subject aged over 40), diminishing the possibility that the findings could be explained by age-related audiological and brain differences.

To estimate the direction of information flow, pairwise path coefficients obtained with SEM were tested (depicted with arrows in **Figure 4**). Directed interactions were found from AC to TPJ [*t*(19) = 8.30, *p* < 0.001], from AC to vPMC [*t*(19) = 2.36, *p* = 0.03], and from vPMC to TPJ [*t*(19) = 2.42, *p* = 0.03]. No significant directionality was found between vPMC and MC [*t*(19) = 0.23, *p* = 0.81].

**FIGURE 5:**
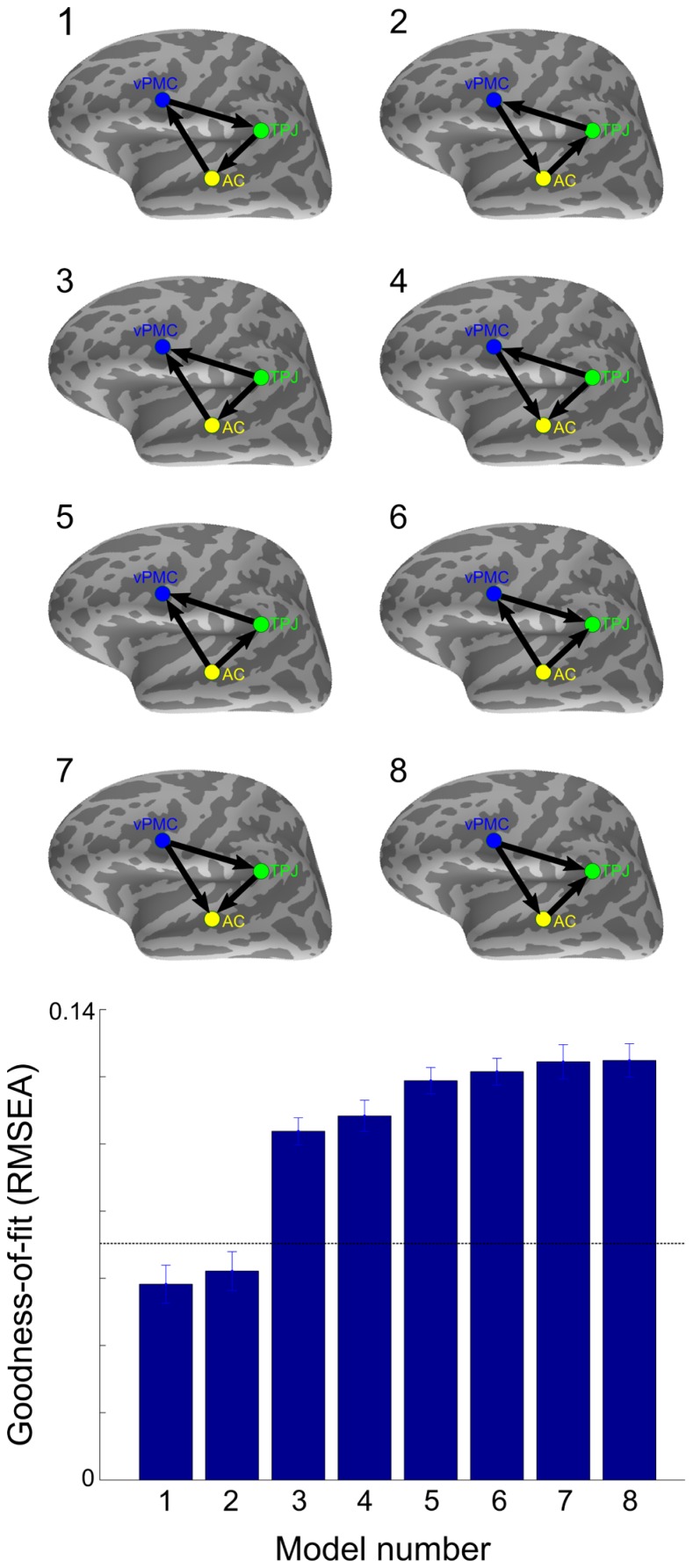
**Comparison between models of effective connectivity**. RMSEA was applied to test the goodness-of-fit between all unidirectional SEM models between the three functionally interconnected left-hemisphere areas. The horizontal dashed line denotes the cut-off point with RMSEA < 0.07 considered a good fit (Steiger, 2007). Error bars indicate SE. AC, auditory cortex; TPJ, temporoparietal junction; vPMC, ventral premotor cortex.

Finally, as shown in **Figure 5**, model comparison was performed between the three functionally interconnected left-hemisphere areas (i.e., AC, TPJ, and vPMC) to determine the model of information flow that best fits the data within the 50–200 ms time window. To avoid the possible bias introduced by comparing models with different degrees of freedom, only unidirectional connections were defined, resulting in a total of 8 candidate models. Two models exhibited mean RMSEA smaller than 0.07, indicating a good fit to the data (Steiger, 2007): AC→vPMC→TPJ→AC (RMSEA: 0.058 ± 0.024; mean ± SD) and AC→TPJ→vPMC→AC (RMSEA: 0.062 ± 0.025; mean ± SD).

## DISCUSSION

The present study examined inter-areal synchrony of neuronal oscillations during speech perception. MEG was recorded while subjects were (1) passively listening to auditory speech sounds (/pa/ and /ta/) presented with or without acoustic noise and (2) engaged in a sensorimotor task involving the identification and overt repetition of the same sounds.

Synchrony between four pairs of left-hemisphere regions showed positive correlation with speech sound identification accuracy within 150 ms after stimulus onset (**Figure 4**). The correlation between AC and TPJ occurred at ~23 Hz and peaked early (60–80 ms). This was followed by correlations between AC and vPMC (90–110 ms at ~20 Hz), TPJ and vPMC (90–120 ms at ~17–23 Hz), and lastly between vPMC and MC (120–140 ms at ~11–14 Hz). *Post hoc* analysis with SEM suggested that information flows from AC to TPJ, from AC to vPMC, and from vPMC to TPJ (**Figure 4**).

These findings suggest that neural communication between auditory speech processing areas and motor cortical areas facilitates phonetic categorization and that the left TPJ functions as an interface where auditory signals are matched with articulatory-motor information. The directed interaction from AC to vPMC and from vPMC to TPJ could be reflecting a processing loop whereby the acoustic speech activates articulatory-motor representations and generates a forward prediction containing information of the sensory consequences of realizing those motor commands. The directed interaction from AC to TPJ between 60–80 ms, on the other hand, could be reflecting a quick sketch of the sensory event (Bar et al., 2006), which is compared against the forward prediction (Rauschecker, 2011). The sensory expectation generated by the forward prediction would then serve to complement the acoustic information for improved phonetic categorization. The SEM model comparison supports the existence of such sensorimotor loops, indicating that models where information flow between the left AC, TPJ, and vPMC forms a loop in either direction fits well to the data (**Figure 5**). This interpretation is in line with the “perception-for-action-control theory” (PACT; Schwartz et al., 2012), according to which speech percepts are shaped by both sensory processing and motor knowledge of speech gestures. As phonetic categorization performance was quantified in the active listening condition involving overt repetition, inherent task differences need to be considered when interpreting the results. However, since access to phonological categories occurs at ~150 ms after sound onset (for a review, see Salmelin, 2007) and since the observed effects occurred within 150 ms after sound onset, it is unlikely that they are reflecting speech preparation (e.g., mental rehearsal) while waiting for the appearance of the visual cue to overtly repeat.

The present results are consistent with our earlier study (Alho et al., 2012), in which positive correlation was found between syllable identification accuracy and PMC response amplitudes at ~100 ms after stimulus onset. These results, along with the findings of another recent study (Szenkovits et al., 2012), suggest also that PMC recruitment varies across subjects, which can be due to individual differences in, e.g., phonological short-term memory (Seghier and Price, 2009). Consistently, Chevillet et al. (2013) found that subjects with more category-selective PMC representations (as observed using fMRI rapid adaptation paradigm) were better able to categorize phonemes in a behavioral test after scanning, thus implying that the representation might be recruited to assist explicit phonetic categorization. The observed individual differences in speech sound identification accuracy can also be explained by differences in allocation of attention. It is noteworthy, however, that selective attention and forward prediction in sensorimotor integration might be supported by similar neural mechanisms. Indeed, the sensory expectation generated by the forward prediction can be understood as increased gain for processing, or reshaping of neuronal receptive fields to be more selective to, the attended/expected auditory features (Hickok et al., 2011). The mechanism for sensorimotor integration could thus, similarly to selective attention, induce short-term plasticity effects on the AC (for a review, see Jääskeläinen and Ahveninen, 2014), and therefore enhance behavioral performance, such as sound discrimination (Kauramäki et al., 2007; Ahveninen et al., 2011). Relatedly, a recent study demonstrated, by using TMS and MEG, that when speech sounds were attended, the articulatory-motor cortex contributed to the auditory processing of the sounds already at 60–100 ms after sound onset, whereas when unattended, the contributing effect started considerably later, at ~170 ms after sound onset (Möttnen et al., 2014). These findings suggest that, although the motor contribution to speech processing seems to occur automatically (Chevillet et al., 2013; Möttönen et al., 2013), early sensorimotor interactions are dependent on attention.

Notably, the phase synchrony effects among AC, TPJ, and vPMC occurred in the beta frequency band (~20 Hz), which is compatible with previous studies revealing an association between beta-band synchrony and sensorimotor integration (for a review, see Siegel et al., 2012). Furthermore, as successful speech perception requires temporal integration of information with high modulation frequency (e.g., formant transitions in /pa/ vs. /ta/), it can be argued that the brain oscillations involved in such a cognitive process must correspond to this frequency (Giraud and Poeppel, 2012). Beta-band oscillations could therefore be sufficiently rapid for the coordination among anatomically distributed neuronal assemblies during encoding and integration of speech information.

Complementing the correlational findings, ANOVA showed a main effect of intelligibility (i.e., noisy vs. clear speech) with stronger synchrony first between left AC and vPMC and later between left TPJ and dPMC for noisy compared to clear speech. Such increase in neural synchrony between auditory and motor regions appears compatible with previous fMRI studies showing a stronger recruitment of motor regions in case of ambiguous stimuli, as e.g., during masked or distorted vs. intelligible speech or during auditory identification of non-native vs. native phonemes (Binder et al., 2004; Callan et al., 2004; Wilson and Iacoboni, 2006; Zekveld et al., 2006). This finding, together with the strong intelligibility x task interaction between left AC and vPMC (caused by enhanced synchrony for noisy compared to clear stimuli only during passive listening; **Figure 3C**) suggests that frontal motor areas support the sensory processing of degraded speech automatically, in the absence of tasks or explicit attention directed to the speech sounds (although, see Wild et al., 2012). As information flow was estimated from AC to vPMC and from dPMC to TPJ, the results converge with findings demonstrating a mediating effect of top-down feedback in the disambiguation of speech (e.g., Gow and Segawa, 2009). The main effect of task (i.e., active vs. passive listening) provided evidence for stronger synchrony between TPJ and vPMC in both hemispheres during active compared to passive perception task, which is likely reflecting enhanced sensorimotor integration (i.e., mapping between auditory and articulatory-motor representations) when people are actively engaged in a speech decision task with subsequent oral responses. This finding is concordant with a recent study showing bilateral sensorimotor transformations during perception in an overt speech repetition task (Cogan et al., 2014) and another showing that while passive listening to speech involved only temporal areas, active speech comprehension was recruiting also bilateral inferior frontal areas (Yue et al., 2013). The left-hemisphere connection showed directionality from vPMC to TPJ, possibly reflecting the integration of motor knowledge with speech inputs through top-down predictions (or attentional modulation, as previously discussed).

In conclusion, our results showed that (1) engagement in a sensorimotor task involving speech sound identification and overt repetition enhanced connectivity bilaterally between the TPJ and vPMC within 200 ms after sound onset; (2) passive listening to noisy speech elicited stronger connectivity than clear speech between left AC and vPMC at ~100 ms, and between left dPMC and TPJ at ~200 ms; and (3) connectivity strength among left AC, vPMC, and TPJ correlated positively with speech sound identification accuracy. The estimated directions of information flow support the idea that top-down feedback from the articulatory-motor areas influences low-level phonetic processing. Taken together, these findings suggest that sensorimotor integration mediates the categorization of incoming speech sounds through reciprocal auditory-to-motor and motor-to-auditory projections.

## Conflict of Interest Statement

The authors declare that the research was conducted in the absence of any commercial or financial relationships that could be construed as a potential conflict of interest.
